# Clinical impact of rare variants associated with inherited channelopathies: a 5-year update

**DOI:** 10.1007/s00439-021-02370-4

**Published:** 2021-09-21

**Authors:** Georgia Sarquella-Brugada, Anna Fernandez-Falgueras, Sergi Cesar, Elena Arbelo, Mónica Coll, Alexandra Perez-Serra, Marta Puigmulé, Anna Iglesias, Mireia Alcalde, Marta Vallverdú-Prats, Victoria Fiol, Carles Ferrer-Costa, Bernat del Olmo, Ferran Picó, Laura Lopez, Ana García-Alvarez, Paloma Jordà, Coloma Tiron de Llano, Rocío Toro, Simone Grassi, Antonio Oliva, Josep Brugada, Ramon Brugada, Oscar Campuzano

**Affiliations:** 1 Medical Science Department, School of Medicine, University of Girona, C/ Emili Grahit 77, 17003, Girona, Catalunya Spain; 2 Arrhythmias Unit, Hospital Sant Joan de Déu, University of Barcelona, Barcelona, Spain; 3 Cardiovascular Genetics Center, University of Girona-IDIBGI, Girona, Spain; 4 Centro de Investigación Biomédica en Red. Enfermedades Cardiovasculares (CIBERCV), Madrid, Spain; 5 Cardiology Service, Hospital Josep Trueta, University of Girona, Girona, Spain; 6 Arrhythmias Unit, Hospital Clinic, University of Barcelona-IDIBAPS, Barcelona, Spain; 7 Medicine Department, School of Medicine, Cádiz, Spain; 8 Institute of Public Health, Section Legal Medicine, Catholic University, Rome, Italy

## Abstract

A proper interpretation of the pathogenicity of rare variants is crucial before clinical translation. Ongoing addition of new data may modify previous variant classifications; however, how often a reanalysis is necessary remains undefined. We aimed to extensively reanalyze rare variants associated with inherited channelopathies originally classified 5 years ago and its clinical impact. In 2016, rare variants identified through genetic analysis were classified following the American College of Medical Genetics and Genomics’ recommendations. Five years later, we have reclassified the same variants following the same recommendations but including new available data. Potential clinical implications were discussed. Our cohort included 49 cases of inherited channelopathies diagnosed in 2016. Update show that 18.36% of the variants changed classification mainly due to improved global frequency data. Reclassifications mostly occurred in minority genes associated with channelopathies. Similar percentage of variants remain as deleterious nowadays, located in main known genes (*SCN5A, KCNH2* and *KCNQ1*). In 2016, 69.38% of variants were classified as unknown significance, but now, 53.06% of variants are classified as such, remaining the most common group. No management was modified after translation of genetic data into clinics. After 5 years, nearly 20% of rare variants associated with inherited channelopathies were reclassified. This supports performing periodic reanalyses of no more than 5 years since last classification. Use of newly available data is necessary, especially concerning global frequencies and family segregation. Personalized clinical translation of rare variants can be crucial to management if a significant change in classification is identified.

## Background

Inherited cardiac channelopathies (ICC) are a group of genetic diseases, principally long QT syndrome (LQTS), Brugada syndrome (BrS), and catecholaminergic polymorphic ventricular tachycardia (CPVT). ICC are characterized by malignant arrhythmias leading to sudden cardiac death (SCD), which may be the first manifestation, especially in the young population (Schwartz et al. 2020). Because ICC are of genetic origin, early identification of deleterious genetic alterations responsible for the diseases can tailor the adoption of preventive management to reduce their lethality (Campuzano et al. 2020a, b). Current guidelines recommend genetic testing of patients diagnosed with an ICC and, if a definite pathogenic variant is identified, also in their relatives who may be genetic carriers and at risk of lethal arrhythmias despite being asymptomatic (Musunuru et al. 2020; Priori and Blomstrom-Lundqvist 2015).

The appropriate interpretation of the pathogenicity of a genetic variant is crucial for translating genetic data into clinical practice. In 2015, the American College of Medical Genetics and Genomics (ACMG) published recommendations for the accurate assessment of rare variants (Richards et al. 2015). These recommendations include analyzing a large number of items, which improves the accuracy but also stringency of classification. In consequence, most rare variants remain of uncertain/ambiguous significance mainly because there is not enough data supporting a definite role in ICC (Ghouse et al. 2018). These ambiguities leave many families affected by ICC with inconclusive genetic diagnoses, which are not helpful in clinical decision-making (Ackerman 2015; Musunuru et al. 2020). In these cases, the ambiguous variant is disregarded in the diagnosis, and only clinical and family history are referenced for risk-assessment and clinical management (Richards et al. 2015). Rare variants of uncertain significance represent an issue also in forensic setting: when they are found in autopsy-negative cases, the implications of this finding is often difficult to expain both to the public authorithies and to the family of the victim (Grassi et al. 2020).

Nowadays, ACMG recommendations are in use and rely on the data available at the moment of genetic data interpretation. However, new data concerning a rare variant may alter its prior classification. Thus, periodic reclassification of rare variants is recommended before clinical translation; as of yet, there is no concrete timeframe for this process. Currently, only a few reports have addressed this novel challenge (Bennett et al. 2019; Campuzano et al. 2020a, b; Chen et al. 2018; Denham et al. 2018; Salfati et al. 2019; VanDyke et al. 2020) despite the impact reclassifications can have improving psychological outcomes and risk stratification while promoting personalized management (Macklin et al. 2019; Tsai et al. 2019). In this study, we updated data to reclassify rare variants associated with the most common ICC, classified 5 years ago following ACMG recommendations, and potential clinical impact.

## Methods

### Cohort

Our retrospective study reanalyzed 49 patients carrying a rare variant classified in 2016 following ACMG recommendations (Richards et al. 2015). All variants were originally interpreted and classified as pathogenic (P), likely pathogenic (LP), or variants of unknown significance (VUS). Variants classified as likely benign (LB) and/or benign (B) in 2016 were not reanalyzed because their global frequencies in 2016 were > 1%, and they had already been reported as non-causative. All rare variants were identified in three groups of ICC: BrS, CPVT, and LQTS. Suspicious cases with an inconclusive diagnosis were not included to avoid bias in the reclassification of genetic variants. Genetic analysis was approved by the ethics committee of Hospital Josep Trueta (Girona, Spain) following the World Medical Association Declaration of Helsinki. Both clinical and genetic data concerning all patients were kept confidential. Written informed consent was obtained from all patients included in the study before genetic analysis.

### Genetic analysis

In 2016, we screened the most prevalent genes associated with SCD, including all then known ICC-associated genes (*ABCC9, ACTC1, ACTN2, AKAP9, ANK2, BAG3, CACNA1C, CACNA2D1, CACNB2, CASQ2, CAV3, CRYAB, CSRP3, DES, DMD, DSC2, DSG2, DSP, EMD, FBN1, FKTN, GLA, GPD1L, HCN4, JPH2, JUP, KCND3, KCNE1, KCNE2, KCNE3, KCNE5, KCNH2, KCNJ2, KCNJ5, KCNJ8, KCNQ1, LAMP2, LDB3, LMNA, MYBPC3, MYH6, MYH7, MYL2, MYL3, MYOZ2, MYPN, NEBL, NEXN, NOS1AP, PDLIM3, PKP2, PLN, PRKAG2, RANGRF, RBM20, RYR2, SCN1B, SCN2B, SCN4B, SCN5A, SGCD, SLMAP, SNTA1, TAZ, TCAP, TGFBR2, TGFB3, TMEM43, TMPO, TNNC1, TNNI3, TNNT2, TP63, TPM1, TRDN, TRPM4, TTN,* and *VCL*). All gene isoforms described in Ensembl 75 (www.ensembl.org/) linked to a RefSeq code (www.ncbi.nlm.nih.gov/refseq/) or CCDS (www.ncbi.nlm.nih.gov/CCDS/). A biotinylated cRNA probe solution was used as a capture probe (Agilent Technologies).

Secondary bioinformatic analysis included adaptor and low-quality base trimming of the FASTQ files. Trimmed reads were mapped with GEM III. The output was sorted, and uniquely and properly mapped read pairs were selected. Finally, variant calling from the cleaned BAM files was performed with SAMtools v.1.2 and an ad hoc developed script. The final annotation steps provided information included in public databases. Non-common (minor allele frequency—MAF- < 1%) genetic variants identified by NEXT GENERATION SEQUENCING (NGS) were confirmed by Sanger sequencing. The exons and exon–intron boundaries of each gene were amplified in both directions. All original sequences obtained in 2016 were comprehensively reanalyzed with updated software (SeqScape v2.7, Applied Biosystems) to detect any alterations not previously identified. No additional rare varinats were identified after comprehensive new reanalysis.

In 2016, identified variations were compared with DNA sequences from 350 healthy Spanish individuals (individuals not related to any index case and of the same ethnicity—Caucasian) as control cases, contrasted with HapMap (www.hapmap.ncbi.nlm.nih.gov/), the 1000 genomes project (www.1000genomes.org/), and the Exome Variant Server—EVS—(www.evs.gs.washington.edu/EVS/); nowadays, all varinats were contrasted in the Genome Aggregation Database—gnomAD—(www.gnomad.broadinstitute.org/). Sequence variants were described following the HGVS rules (www.hgvs.org/) and checked in Mutalyzer (www.mutalyzer.nl/). Finally, nowadays all rare variants were consulted in ClinGen (www.clinicalgenome.org/), VarSome (www.varsome.com/), the SCD-associated Variants Annotation Database—SVAD—(www.svad.mbc.nctu.edu.tw/), CardioClassifier (www.cardioclassifier.org/), InterVar (www.wintervar.wglab.org/), CardioVAI (www.cardiovai.engenome.com/), Franklin (www.franklin.genoox.com/clinical-db/home), and CardioBoost (www.cardiodb.org/cardioboost/).

### Data

An exhaustive review of the literature concerning each variant was performed through February 2021. Data was collected from: HGMD (www.hgmd.org), ClinVar (www.ncbi.nlm.nih.gov/clinvar/intro/), the National Center for Biotechnology Information SNP database (www.ncbi.nlm.nih.gov/SNP), Index Copernicus (www.en.indexcopernicus.com), Google Scholar (www.scholar.google.es), Springer Link (www.link.springer.com), Science Direct (www.sciencedirect.com), the Excerpta Medica Database (www.elsevier.com/solutions/embase-biomedical-research), and the IEEE Xplore Digital Library (www.ieeexplore.ieee.org/Xplore/home.jsp).

### Classification

Five years ago, variants were classified following ACMG standards and guidelines, the same standards currently in use.(Richards et al. 2015) It is important to note that in last years some updates have been included in this guidelines: the PM2 item in the ACMG classification was considered fulfilled if the MAF in relevant population databases was ≤ 0.1% (Lek et al. 2016). The vast majority of reported pathogenic variants in ICC are extremely rare (MAF < 0.01%) (Kobayashi et al. 2017). The classification ‘high degree of pathogenicity’ (item PVS1) should only be used for rare variants in genes where loss of function is a well-established disease mechanism.(Abou Tayoun et al. 2018) Genetic data were independently evaluated and classified by five experts in the genetics of ICC (three cardiologists and two geneticists). All investigators discussed and agreed on a final classification of all variants to avoid bias.

## Results

### Cohort

**Fig. 1:**
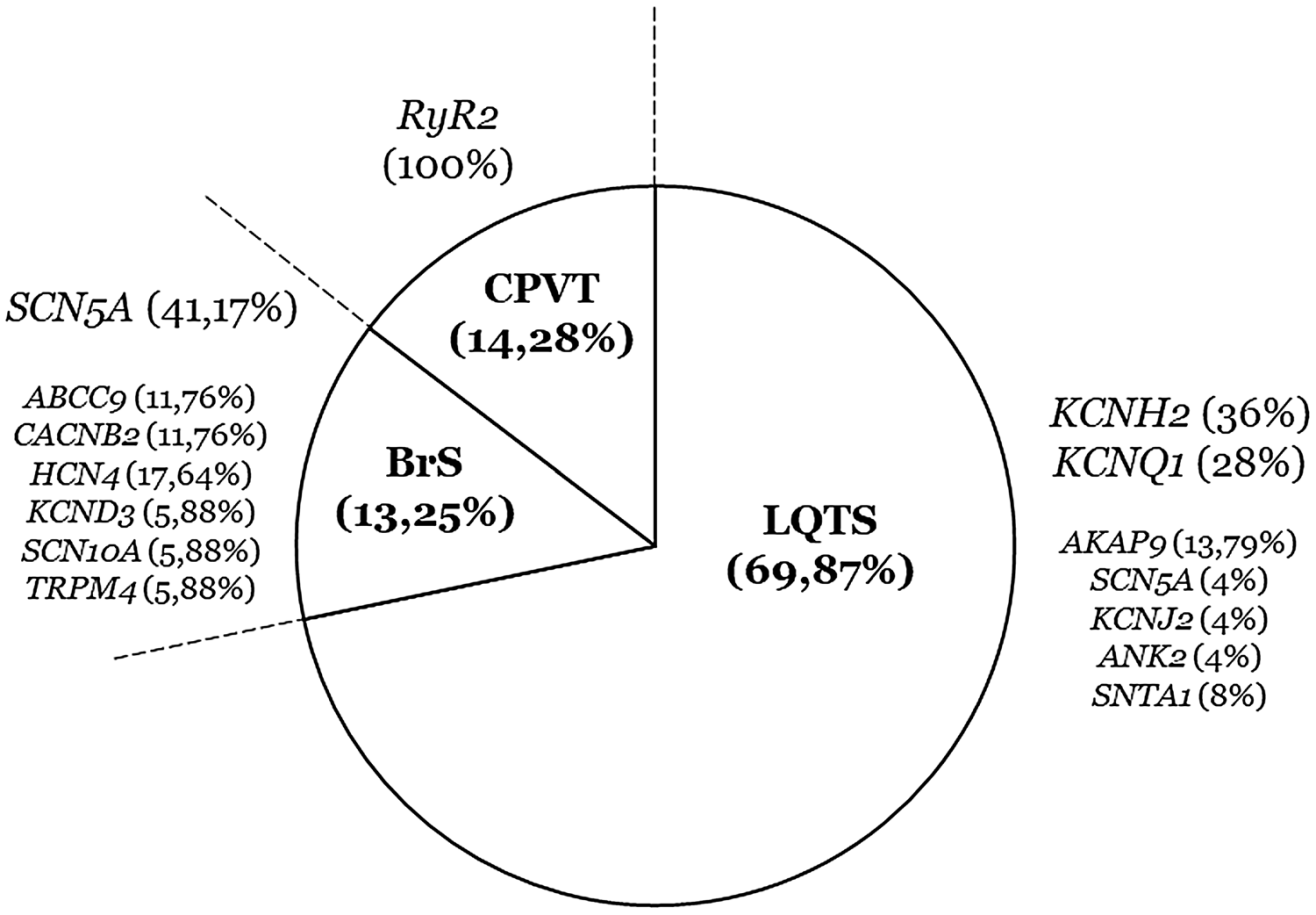
Population of study. A total of 49 cases were reanalysed, mostly diagnosed with LQTS (69.87%). In each disease, rare variants classified as pathogenic and likely pathogenic in 2016. *BrS* Brugada Syndrome, *CPVT* catecholaminergic polymorphic ventricular tachycardia, *LP* likely pathogenic, *LQTS* long QT syndrome, *P* pathogenic

Our retrospective study included 49 cases, all Caucasian (73.46% men). Ages ranged from 21 to 64 years (mean age: 42.2 years). The cohort included 25 cases of LQTS (51.02%), 17 cases of BrS (34.69%), and seven cases of CPVT (14.28%) (Fig. 1). Review of clinical data did not change the definite diagnostic of ICC from 5 years ago in any case.

### Genetics

Each case included in our study carried one rare variant reported in 2016 as potentially associated with diagnosed ICC. These 49 rare variants were localized in 14 genes encoding ion channels or associated proteins: *ABCC9* (two in BrS), *AKAP9* (four in LQTS), *ANK2* (one in LQTS), *CACNB2* (two in BrS), *HCN4* (three in BrS), *KCND3* (one in BrS), *KCNH2* (nine in LQTS), *KCNJ2* (one in LQTS), *KCNQ1* (seven in LQTS), *RyR2* (seven in CPVT), *SCN10A* (one in BrS), *SCN5A* (seven in BrS and one in LQTS), *SNTA1* (two in LQTS), and *TRPM4* (one in BrS) (Fig. 1). Forty-seven identified variants were exonic (95.92%), 40 were *missense* and seven radical (two nonsense and five indels) (Table 1). Two intronic variants (4.08%) were identified in two LQTS cases; curiosly, one intronic variant was identified in homozygosis (c.604 + 1G > C_*KCNQ1*).

**Table 1:** Genetic data of variants

Case	Diagnosis	Gene	Nucleotide	Protein	dbSNP	gnomAD	ACMG 2016	ACMG 2021
1	BrS	*ABCC9*	c.2050G>A	(p.Gly684Ser)	rs148174226	210/250.964 (0.08%)	VUS	LB
2	BrS	*ABCC9*	c.4603G>A	(p.Ala1535Thr)	NA	NA	VUS	VUS
3	BrS	*CACNB2*	c.641G>C	(p.Ser214Thr)	rs149253719	251/250.608 (0.1%)	VUS	LB
4	BrS	*CACNB2*	c.1702G>A	(p.Val568Ile)	rs142639223	61/251.242 (0.02%)	VUS	LB
5	BrS	*HCN4*	c.979A>G	(p.Ile327Val)	NA	NA	VUS	VUS
6	BrS	*HCN4*	c.2864C>T	(p.Pro955Leu)	rs371562763	7/149.646 (0.004%)	VUS	VUS
7	BrS	*HCN4*	c.3502_3505delTTTG	(p.Phe1168GlyfsTer12)	rs786205259	26/240.632 (0.01%)	VUS	LB
8	BrS	*KCND3*	c.1649G>A	(p.Arg550His)	rs151164490	12/251.460 (0.004%)	VUS	VUS
9	BrS	*SCN10A*	c.2972C>T	(p.Pro991Leu)	rs138413438	242/250.924 (0.09%)	VUS	LB
10	BrS	*SCN5A*	c.664C>T	p.Arg222Ter	rs794728849	NA	LP	P
11	BrS	*SCN5A*	c.736T>C	(p.Ser246Pro)	NA	NA	VUS	VUS
12	BrS	*SCN5A*	c.2302A>G	(p.Ile768Val)	NA	NA	VUS	VUS
13	BrS	*SCN5A*	c.4183T>C	(p.Tyr1395His)	NA	NA	VUS	VUS
14	BrS	*SCN5A*	c.4372C>T	(p.Arg1458Trp)	rs137854602	14/251.272 (0.005%)	LP	LP
15	BrS	*SCN5A*	c.4339C>G	(p.Leu1447Val)	rs199473266	5/251.446 (0.01%)	LP	LP
16	BrS	*SCN5A*	c.4906G>A	(p.Asp1636Asn)	rs1060499900	1/251.488 (0.0003%)	LP	LP
17	BrS	*TRPM4*	c.2561A>G	(p.Gln854Arg)	rs172155862	159/197.334 (0.08%)	VUS	LB
18	CPVT	*RYR2*	c.314C>T	(p.Ala105Val)	NA	NA	VUS	VUS
19	CPVT	*RYR2*	c.3038G>A	p.Arg1013Gln	rs149514924	117/249.160 (0.04%)	VUS	VUS
20	CPVT	*RYR2*	c.6352A>G	p.Asn2118Asp	NA	NA	VUS	VUS
21	CPVT	*RYR2*	c.7807G>A	(p.Ala2603Thr)	rs59331340	22/197.538 (0.01%)	VUS	VUS
22	CPVT	*RYR2*	c.11989A>G	(p.Lys3997Glu)	rs1064794210	NA	VUS	VUS
23	CPVT	*RYR2*	c.12919C>T	(p.Arg4307Cys)	rs200092869	86/248.746 (0.03%)	VUS	VUS
24	CPVT	*RYR2*	c.14302G>A	(p.Val4768Ile)	rs775534249	4/249.196 (0.001%)	VUS	VUS
25	LQTS	*AKAP9*	c.4707T>G	(p.Ile1569Met)	rs1207821150	1/249.300 (0.0004%)	VUS	VUS
26	LQTS	*AKAP9*	c.8949G>T	(p.Glu2983Asp)	NA	NA	VUS	VUS
27	LQTS	*AKAP9*	c.9102C>G	(p.Phe3034Leu)	NA	NA	VUS	VUS
28	LQTS	*AKAP9*	c.9689A>G	(p.Lys3230Arg)	rs192845338	59/251.432 (0.02%)	VUS	LB
29	LQTS	*ANK2*	c.84+5A>G	NA	NA	NA	VUS	VUS
30	LQTS	*KCNH2*	c.214_215delCCinsGG	(p.Pro72Gly)	NA	NA	LP	LP
31	LQTS	*KCNH2*	c.221_242del	(p.Thr74ArgfsTer35)	rs1389503709	NA	LP	LP
32	LQTS	*KCNH2*	c.242A>C	(p.Gln81Pro)	NA	NA	VUS	VUS
33	LQTS	*KCNH2*	c.422C>T	(p.Pro141Leu)	rs199472864	79/251.238 (0.03%)	VUS	VUS
34	LQTS	*KCNH2*	c.1501G>A	p.Asp501Asn	rs199472912	NA	P	P
35	LQTS	*KCNH2*	c.1525G>A	(p.Asp509Asn)	rs370637245	1/251.370 (0.0003%)	LP	LP
36	LQTS	*KCNH2*	c.1681G>A	p.Ala561Thr	rs199472921	NA	P	P
37	LQTS	*KCNH2*	c.2230C>T	(p.Arg744Ter)	rs189014161	NA	LP	LP
38	LQTS	*KCNH2*	c.3067C>G	(p.Leu1023Val)	NA	NA	VUS	VUS
39	LQTS	*KCNJ2*	c.1229A>G	(p.Asn410Ser)	rs141069645	86/251.146 (0.03%)	VUS	LB
40	LQTS	*KCNQ1*	c.556G>A	(p.Gly186Ser)	rs199473398	NA	LP	LP
41	LQTS	*KCNQ1*	c.757_758delTCinsAA	(p.Ser253Asn)	NA	NA	LP	LP
42	LQTS	*KCNQ1*	c.604 + 1G>C (HM)	NA	NA	NA	VUS	VUS (HM)
43	LQTS	*KCNQ1*	c.935C>T	p.Thr312Ile	rs120074182	NA	P	P
44	LQTS	*KCNQ1*	c.1022C>T	(p.Ala341Val)	rs12720459	NA	LP	LP
45	LQTS	*KCNQ1*	c.1486_1487delCT	(p.Leu496AlafsTer19)	rs397508090	NA	LP	LP
46	LQTS	*KCNQ1*	c.1896A>T	(p.Arg632Ser)	NA	NA	VUS	VUS
47	LQTS	*SCN5A*	c.4493A>T	(p.Gln1498Leu)	rs1387460395	1/249.338 (0.0004%)	VUS	VUS
48	LQTS	*SNTA1*	c.40G>A	(p.Glu14Lys)	rs786205846	8/40.454 (0.01%)	VUS	VUS
49	LQTS	*SNTA1*	c.160G>C	(p.Gly54Arg)	rs786205848	8/65.264 (0.01%)	VUS	VUS

*ACMG* American College of Medical Genetics and Genomics, *B* benign, *BrS* Brugada syndrome, *CPVT* catecholaminergic polymorphic ventricular tachycardia, *LB* likely benign, *LQTS* long QT syndrome, *LP* likely pathogenic, *NA* not available, *P* pathogenic, *VUS* variant of uncertain significance

**Fig. 2:**
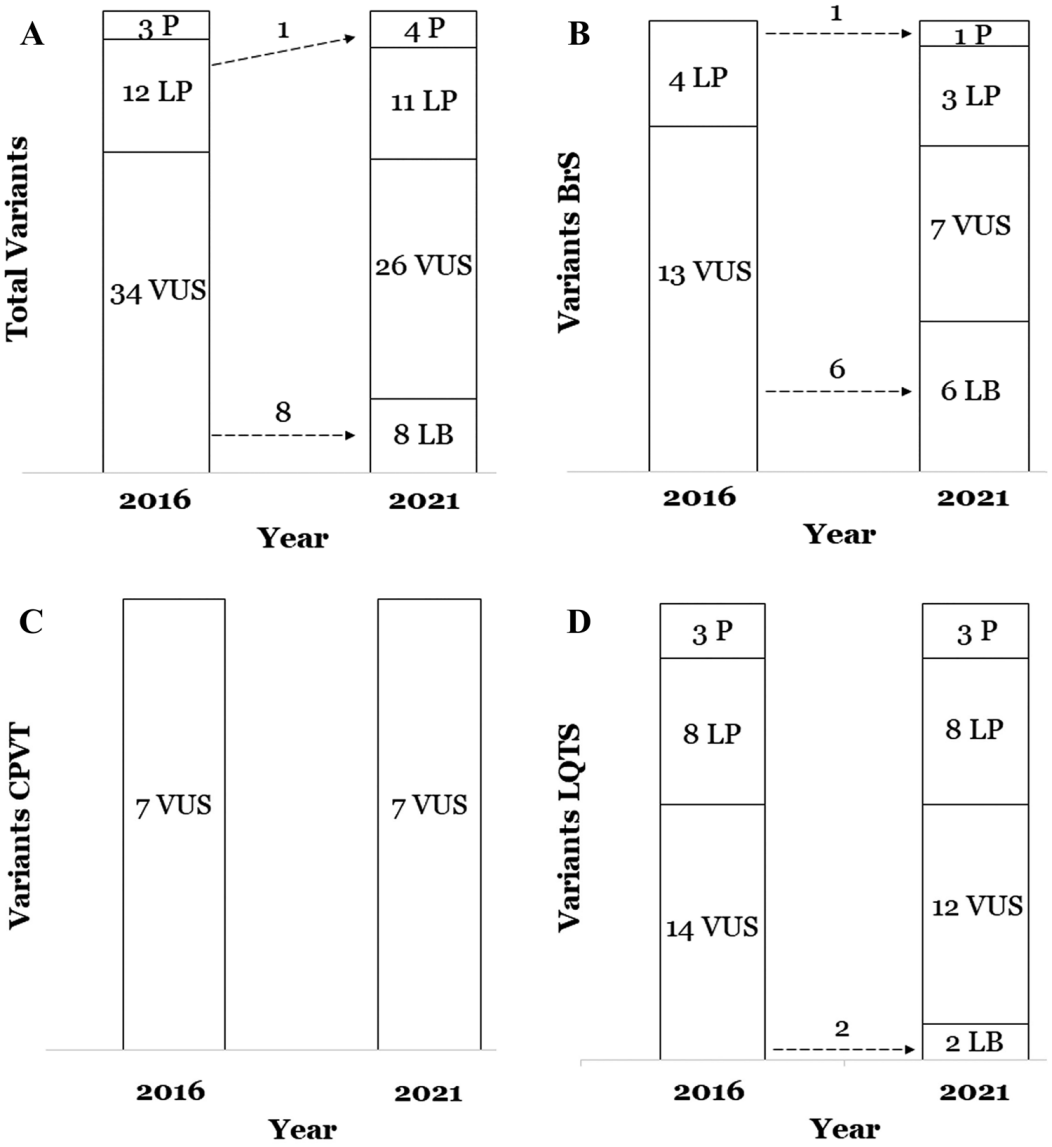
Reclassification of rare variants after 5 years. **A** Global reanalysis of all variants. **B** Reanalysis in Brugada syndrome cases. **C** Reanalysis in Catecholaminergic Polymorphic Ventricular Tachycardia cases. **D** Reanalysis in Long QT syndrome cases. *B* benign, *BrS* Brugada syndrome, *CPVT* catecholaminergic polymorphic ventricular tachycardia, *LB* likely benign, *LP* likely pathogenic, *LQTS* long QT syndrome, *P* pathogenic, *VUS* variant of uncertain significance

The classification performed in 2016 concluded that there were: 3 P variants (6.1%)—all in 3 LQTS cases, 12 LP variants (24.48%)—4 in BrS and 8 in LQTS, and 34 VUS (69.38%)—13 in BrS, 7 in CPVT, and 14 in LQTS (Table 1). Current reanalysis revealed significant changes in the classification of 18.36% (9 of 49) of the rare variants (Fig. 2, Table 2). Classifications based on all newly identified available data yielded: 4 P variants (8.16%)—1 in BrS and 3 in LQTS, 11 LP variants (22.44%)—3 in BrS and 8 in LQTS, 26 VUS variants (53.06%)—7 in BrS, 7 in CPVT, and 12 in LQTS, and 8 LB variants (16.32%)—6 in BrS, and 2 in LQTS (Fig. 2) (Table 1). The previous classifications of 8 rare variants were downgraded from VUS to LB, and one was upgraded from LP to P (in BrS, p.Arg222Ter_*SCN5A*). The percentage of P variants was similar in 2016 and now (6.1% and 8.16%, respectively), and also concerning LP (24.48% in 2016 and 22.44% nowadays). Concerning VUS, there was the predominant group in 2016 and also now; however, a decrease of 16.32% occurred, from 34 VUS variants (69.38%) in 2016 to 26 VUS variants (53.06%) nowadays (Fig. 2A, Table 1). All these VUS variants changed to LB due to update in global population frequencies (Table 2).

**Table 2:** Ratio changes and disease

Variants	Changes	Disease/changes	Cause of change	
			Frequency	New data
49	9 (18.36%)	BrS / 7 (14.28%)	6 (12.24%)	1 (2.04%)
		CPVT / 0 (0%)	–	–
		LQTS / 2 (4.08%)	2 (4.08%)	–

“New data” refers to other novel data available nowadays different to frequency in population (in silico prediction, in vitro studies, or any available study in a large family diagnosed)

*BrS* Brugada syndrome, *CPVT* catecholaminergic polymorphic ventricular tachycardia, *LQTS* long QT syndrome

Specifically, seven BrS rare variants changed (14.28%): six VUS to LB -all in minor genes- (Fig. 2B). As mentioned, changes from VUS to LB occurred due to new variant frequencies in population. The remain modification in BrS cohort refer to one LP variant changed to P (p.Arg222Ter_*SCN5A*) due to new data published in last 5 years (Fig. 2, Table 2). In CPVT cases, no changes were performed. All rare variants were identified in *RyR2* and classified as VUS in 2016 and now (Fig. 2C). Finally, in LQTS, only two variants changed (4.08%), concretely from VUS to LB (Fig. 2D). The causes of these changes were mainly a high MAF reported in new global frequency data (Fig. 2, Table 2).

## Discussion

Genetic analysis of a diagnosed ICC is highly recommended to unravel the cause of the disease (Priori and Blomstrom-Lundqvist 2015). A high percentage of patients carrying a deleterious genetic variant remains asymptomatic; thus, early identification of a genetic alteration definitively associated with the disease may drive therapeutic pharmacological and non-pharmacological management to reduce risk of lethal arrhythmias (Ackerman et al. 2011; Campuzano et al. 2020a, b; Musunuru et al. 2020). However, a main challenge for the clinical translation of genetic data is understanding the definite role of a rare variant. Misinterpretation of rare variants may lead to inaccurate genetic diagnoses and/or the adoption of unnecessary and/or inappropriate preventive and therapeutic approaches.

Currently, the classification of a rare variant is performed following the ACMG recommendations and according to data available at the moment of interpretation (Richards et al. 2015). A geneticist who misinterprets the significance of a variant can be considered legally liable only if the current scientific evidence and the application of these criteria whould have permitted a correct classification (Grassi et al. 2020). However, a misinterpretation is often due to the lack of sufficient scientific evidence. Continuous development of clinical data, bioinformatic tools and advances in genomic knowledge highlights the critical need for periodic revision (Salfati et al. 2019). In addition, publication of new data may also alter the prior classification of a rare variant, but there is, as yet, no concrete timeframe for reanalysis. Our study shows that nearly 20% of overall variants changed classification within 5 years, suggesting a *lustrum* should be a potential time for reanalysis, at least for rare variants associated with main ICC. However, identifying the optimal time period between reclassifications requires the assessment of several interval sizes in large cohorts.

### Impact of reanalysis

The same number of rare variants with a deleterious/potentially deleterious role were identified in 2016 and now (30.61%), and all located in main genes associated with ICC (*SCN5A, KCNQ1* and *KCNH2*). One variant upgraded from LP to P, accordingly to a recent manuscript by Harrison et al. that found that LP variants became P between 2016 and 2019 (Harrison and Rehm 2019). However, the results of the Harrison et al*.* study were based on variant modifications identified only in ClinVar, with the authors being uncertain that all classifications were according to ACMG guidelines. The authors did state that most radical variants, i.e., those leading to premature truncation of proteins and/or frameshifts, should be considered highly damaging if not identified at high frequency in population and, therefore, should be carefully analyzed. Our results concord with this point as the majority of indels in our study remained classified as LP except one, showing high frequency in global population and classified as LB. Therefore, for each patient, *missense* rare variants should be comprehensively analyzed considering all available data to be properly prioritized in a personalized clinical context (Eilbeck et al. 2017).

In the case of VUS, a rare variant classified as ambiguous does not provide molecular confirmation of a diagnosis but cannot be discarded as indicating a low risk of malignant arrhythmias for any patient, at least until additional data clarifies its clinical role (Musunuru et al. 2020). In our study, most rare variants were classified as VUS in both periods of analysis (from 69.38 to 53.06%, currently). All VUS changed to LB due to substantial increase of MAF seen with ongoing analysis of the global population. This point notes the key role of global frequencies and its correlation with the prevalence of the ICC in the population. Thus, reducing uncertainty by excluding the potential effect of some VUS variants, at least on ICC, is probably one of the remarkable benefits of reanalysis. These results accord with those of previous studies showing VUS rarely change to P or LP variants (Lahrouchi et al. 2017). However, until the role of a rare variant can be certainly or likely assessed, no inference should be made on its possible significance and no impact in clinical management of the patient can be justified. Therefore, clinical translation of VUS should be performed with caution and families counselled regarding the current limitations of reliable clinical interpretation. In our opinion, a complete family segregation together with an accurate global frequency are the most robust tools to distinguish a potential damaging variant from other rare variants, and free access to available frequency databases is a quick and crucial step. Other data, e.g., functional studies, may corroborate the pathogenic role of a rare variant (Glazer et al. 2020), but unfortunately, a complete family segregation as well as functional analysis are not available for most rare variants currently associated with ICC. It is also important to note that a low percentage of current VUS may eventually be shown to confer a real risk of ICC (Blekhman et al. 2008); however, distinguishing pathogenic VUS from the majority of VUS is one of the main current challenges in the ICC field (Cherny et al. 2020; Tsai et al. 2019).

The ACMG recommends how variants should be classified, but there is no consensus for how often reclassification should occur. Therefore, reanalysis of rare variants occurs mainly due to a clinician’s request, identification of a previously classified variant in a new patient, or other new data (El Mecky et al. 2019). Smith et al. reported that 3% of ICC rare variants became reclassified after 1 year (Smith et al. 2017). In another study, reclassification after 7 years (from 2011 to 2018) increased the molecular diagnosis of ICC by 2% in cases of unexpected decease (Salfati et al. 2019). In an additional cohort analyzed for genetic arrhythmia and cardiomyopathy disorders from 2006 to 2017, 22% of variants changed classification and approximately 10% changed in a way that altered clinical interpretation (Cherny et al. 2020). Following similar results, VanDyke et al. published that 35% of the variants associated with ICC had classifications that differed from their firsts reports (all prior 2015) (VanDyke et al. 2020). Therefore, it is widely accepted that variants did not classify following ACMG recommendations should be updated immediately due to the potential clinical impact associated (Campuzano et al. 2020a, b). In addition, it is also accepted that periodic reinterpretation of variants is necessary for clinical management of patients with ICC but remains undefined how often a reanalysis is necessary if variants are already classified following ACMG recommendations. Our study provides first evidence that a 5-year timeframe is adequate to manage the rapid obsolescence of genetic data interpretations, at least in ICC despite further investigations should be performed in large cohorts.

### Brugada syndrome

In 2019, Denham et al. reported that only 37% of rare variants previously considered deleterious for BrS were definitely reclassified as P or LP following ACMG guidelines (Denham et al. 2018). Accordingly, in our study 23.52% of BrS rare variants were classified as deleterious in 2016, being currently the same percentage classified with a harmful role. All these rare deleterious variants were located in *SCN5A*. The number of VUS was reduced since 2016 (from 13 to 7), 6 to LB (in minor genes) and only one to LP (in *SCN5A*). Taking all these data into account, our study reinforce *SCN5A* as the major gene associated with BrS (Walsh and Wilde 2019).

### Catecholaminergic polymorphic ventricular tachycardia

In our cohort of CPVT, all identified rare variants were located in *RyR2*, reinforcing the role of this gene as the major player in CPVT. A recent study reclassified all *RYR2* variants using ACMG recommendations and found that only 7.8% of previously disease-associated variants remained as LP/P (Olubando et al. 2020). Another study published in 2018 found that five upgraded from VUS to LP/P and six downgraded from P/LP to VUS when ACMG recommendations were applied (Roston et al. 2018). The same trend was observed in a cohort of children in which two out of five (40%) VUS in *RYR2* upgraded to LP/P using ACMG recommendations (Bennett et al. 2019). This supports an assertion made in prior studies where an urgent reanalysis of rare variants associated with ICC should be done if they were not classified originally following ACMG recommendations (Campuzano et al. 2020a, b; VanDyke et al. 2020). The number of VUS in our cohort of CPVT cases did not change from 2016 to date. In comparison to BrS and LQTS, the estimated prevalence of CPVT is much lower; this could be the main reason that less clinical and functional data have been published in last 5 years.

### Long QT syndrome

In our cohort of LQTS, the same three P rare variants remained after 5 years, making up 12% of the total number of LQTS variants. Of the LP variants, 8 (100%) retained their classification. In a study focused on a cohort of children diagnosed with LQTS, 66.66% of VUS were reclassified as LP/P (Bennett et al. 2019). In contrast, another study reported 14.3% of variants previously classified as LP/P were downgraded to VUS (Westphal et al. 2020). This ambiguity reinforces the need for additional studies focused on a proper reinterpretation, especially if variants were not originally classified following ACMG recommendations (Campuzano et al. 2020a, b; VanDyke et al. 2020). In our cohort, LP/P rare variants remain mainly located in *KCNH2*, and *KCNQ1*, consistent with recent critical reappraisals of genes implicated in LQTS (Adler et al. 2020; Giudicessi et al. 2018). Concerning VUS, we identified two rare variants were reclassified to LB due to new available MAF, supporting periodic checking of available frequency data to clarify the role of ambiguous variants.

### Clinical translation

The main aim of a comprehensive genetic interpretation is clinical translation. Clinical features and phenotypic context are critical for effectively interpreting the clinical impact of rare variants in ICC, helping to improve the diagnosis, treatment, and family screening of patients who currently receive uncertain genetic test results (Walsh et al. 2021). A recent study focused on the reclassification of VUS in ICC concluded that disease-specific phenotypes significantly increase the accuracy of classification and reinforce the need for clinical data in genetic diagnoses, aiding variant interpretation (Bennett et al. 2019). Hence, updating the classification of a rare variant may have significant clinical impact on patients and their relatives. Therapeutic management can be modified, but emotional and psychological impacts may have lasting effects (Vears et al. 2018). Another important point is the economic impact of reinterpretation. There is currently no legal duty for laboratories to reanalyze data neither in clinical nor in forensic cases (Giesbertz et al. 2019). Hence, the real cost and who pays for the service of reassessing genetic variants over time remains unclear (Vears et al. 2020). A reinterpretation can lead to recommendations for preventive measures and health care treatments that were not considered to be necessary until after reanalysis (Pagan et al. 2020). Despite this, there are no current guidelines informing the cost-effectiveness of reanalysis.

Finally, it is important to clarify that new genetic data should be discussed by a group of experts in ICC (Muller et al. 2020) and, to avoid misinterpretation, an expert cardiologist in genetics should explain to patients what reclassification entails (Bombard et al. 2019; Burns et al. 2018). These expectations should be explicitly delineated as part of the informed consent process before a sample is obtained and reviewed again when disclosing initial results (David et al. 2019). From our point of view, care of ICC families should follow the ethical premise that improving medical care should be the major reason for establishing clinical or research guidelines.

Our study has some limitations. First, variant classification is subject to inherent intra- and inter-laboratory differences in data interpretation (Amendola et al. 2016). To minimize this in our study, five of the authors (OC, GSB, EA, AF, and RB) performed independent classification following ACMG recommendations, and all authors included in the authorship came to a consensus regarding the final classification decision. Despite this fact, we assume that some variants may be interpreted in a different way in other laboratories, especially concerning VUS variants. Second, concerning genetic diagnosis, it should be noted that patients may carry additional rare variants in ICC-related genes that are currently unknown and hence not included in our gene panel. Third, it is important to consider that our cohort was limited and comprehensive reassessment should be performed in larger ICC cohorts to corroborate periodic reclassification. Despite not concrete time, similar studies also recommend periodic reanalysis. We propose, for the first time, a time frame for genetic reanalysis in ICC. Fourth, we have not assessed the economic cost of a comprehensive reinterpretation and who (government, hospital/health institution, or patient) should assume this cost. This is a controverse point that should be deeply analyzed due to each country has a particular health system. Finally, lack of available data for some rare variants included in our study currently impedes more accurate interpretation, remaining as ambigous.

In summary, a comprehensive genetic interpretation of rare variants associated with inherited channelopathies is warranted because it has practical consequences for patients and their relatives. We found that 18.36% of rare variants changed classification, based on the current ACMG criteria, within 5 years. These changes were mainly due to new data on the global frequencies of rare variants. However, many rare variants remain of ambiguous significance due to a lack of functional data and, most importantly, conclusive family segregation. We recommend performing reanalysis of rare variants associated with inherited channelopathies at least every 5 years to incorporate new data concerning pathogenicity despite identifying the optimal time period between reclassifications requires more systematic investigations. When significant changes in classification occur, the cardiologist should promptly inform interested patients and, if necessary, modify the therapeutic approach.

## Data Availability

All data generated or analysed during this study are included in this published article.
